# Pattern and Outcome of Pediatric Traumatic Brain Injury at Hawassa University Comprehensive Specialized Hospital, Southern Ethiopia: Observational Cross-Sectional Study

**DOI:** 10.1155/2020/1965231

**Published:** 2020-01-29

**Authors:** Tuji Bedry, Henok Tadele

**Affiliations:** ^1^Department of Pediatrics and Child Health, College of Health Sciences, Dire Dawa University, Dire Dawa, Ethiopia; ^2^Department of Pediatrics and Child Health, College of Health Sciences, Addis Ababa University, Addis Ababa, Ethiopia

## Abstract

**Background:**

Traumatic brain injury (TBI) is the most common cause of death/disability in children. The Glasgow coma scale and other parameters are used for treatment/follow-up of TBI. Childhood TBI data are scarce from sub-Saharan Africa. The study aimed to determine the pattern and predictors of the TBI outcome in Southern Ethiopia.

**Methods:**

An observational cross-sectional study was conducted from September 2017 to September 2018 at Hawassa University Hospital. Structured questionnaires were used for data collection. Significant associations were declared at a *P* value of <0.05.

**Results:**

There were 4,258 emergency room (ER) visits during the study period, and TBI contributed to 317 (7.4%) cases. The mean age of study subjects was 7.66 ± 3.88 years. Boys, predominantly above 5 years of age, comprise 218 (68.8%) of the study subjects with a male to female ratio of 2.2 : 1. Pedestrian road traffic accidents (RTA), 120 (37.9%), and falls, 104 (32.8%), were the commonest causes of TBI. Mild, moderate, and severe TBI were documented in 231 (72.9%), 61 (19.2%), and 25 (7.9%) of cases, respectively. Most of the TBI cases presented within 24 hrs of injury, 258 (81.4%). Recovery with no neurologic deficit, 267 (84.2%); focal neurologic deficit, 30 (9.5%); depressed mentation, 10 (3.2%); and death, 10 (3.2%), were documented. Signs of increased intracranial pressure (ICP) at admission [AOR: 1.415 (95% CI: 1.4058–9.557)], severe TBI [AOR: 2.553 (95% CI: 1.965–4.524)], presence of hyperglycemia [AOR: 2.318 (95% CI: 1.873–7.874)], and presence of contusion, diffuse axonal injury (DAI), or intracranial bleeding on the head computed tomography (CT) scan [AOR: 2.45 (95% CI: 1.811–7.952)] predicted poor TBI outcome.

**Conclusion:**

TBI contributed to 7.4% of pediatric ER visits. Pedestrian RTA and falls, early presentation (<24 hours of injury), and mild form of TBI among boys were the most common documented patterns. ICP, hyperglycemia, severe TBI, and presence of contusion, DAI, or intracranial bleeding on head CT predicted poor outcome. Strategies to ensure road safety and to prevent falls and animal-related injuries and TBI follow-up for ICP and glycemic controls are recommended.

## 1. Introduction

Traumatic brain injury (TBI) is a brain injury that occurs following a blow to the head, a fall, a bullet, a high-speed crash, or explosion injuries. TBI could be an open (penetrating) or closed type [1]. Childhood injury requires immediate attention given its contribution to high childhood mortality and long-term disabilities. Injuries contribute to 5.4% (265,000–348,000) of childhood deaths per year worldwide [2]. In 2015, injuries resulted in 25,000 deaths among the Ethiopian children 0–14 years of age [3]. TBI is a single, severe, and the most common form of injury in children [4].

Worldwide, it is estimated that TBI affects 69 million individuals each year. Low- and middle-income countries (LMICS) have three times higher TBI burden than high-income countries. Road traffic-related head injuries were reported to be common in LMICS. Globally, TBI is projected to be the third leading cause of death and injury by the World Health Organization in 2020 [2, 5, 6]. Pediatric TBI is reported to be the most common cause of injury-related death, and it commonly follows road traffic accidents and falls [7, 8]. TBI accounted for 8.3% of the pediatric emergency department (ED) visits with mild severity according to the Western studies [9, 10]. Several studies from developing countries documented TBI as a very common public health problem with milder severity and most notably following motor vehicle accidents [11–17]. Road traffic accidents (RTA) and falls from height were common reported pediatric TBI causes. Assaults or intentional injuries were reported in the minority of cases [2, 9–11, 18].

Primary brain injury involves initial tear, shear, or hemorrhage. Secondary injuries, which are targets for interventions, usually involve cascades of biologic reactions following primary injury. These changes include cellular, chemical, tissue, or blood vessel changes in the brain resulting in further damage to the brain tissue [19, 20]. Severity of TBI is assessed using the Glasgow coma scale (GCS) and graded into mild (13–15), moderate (9–12), and severe (≤8). The GCS also assists in assessing the outcome of TBI cases [6, 21, 22]. Mild TBI presents with concussion symptoms, affecting physical, cognitive, and emotional (affective) domains. Various degrees of autonomic and neurologic dysfunctions are seen in moderate and severe TBI cases in addition to mild TBI features [23, 24]. Head computed tomography (CT) is recommended for children presenting with drowsiness or decreased mentation, any sign of basal skull fracture, focal neurologic deficit, etc. [25]. Several arguments are forwarded on abandonment of skull X-ray as an investigation means for TBI [26, 27].

Acute management of TBI includes resuscitation and airway management, nutritional support, intubation when GCS ≤ 8, follow-up for ICP and other complications, and neurosurgical intervention [6]. Presence of cerebral edema, GCS ≤ 8, hypoxemia, and hypernatremia were reported as predictors of poor TBI outcome [21, 22, 28]. Poor TBI outcome was documented in resource-limited settings, and neurocritical protocol for prehospital care was recommended [29]. The TBI mortality rate ranged from 8% in Western settings to 21.2% in the developing regions [7, 11, 29]. Lack of prospective studies and injury data registries in most parts of Africa has made the assessment of TBI difficult [15]. Evidences on the epidemiologic profile and outcome of TBI are also scarce from Ethiopia, sub-Saharan Africa. Our study aimed to determine the epidemiologic profile and outcome of childhood TBI at a tertiary hospital in Southern Ethiopia.

## 2. Methods

### 2.1. Study Area

The study was conducted at Hawassa University Comprehensive and Specialized Hospital (HUCSH), Hawassa, Ethiopia. Hawassa city is located 270 km south of Addis Ababa, Ethiopia's capital. HUCSH is the first and largest referral and teaching hospital in Southern Ethiopia. It serves a catchment population of over 18 million. The pediatrics department provides inpatient and outpatient services. Neurosurgical interventions are provided by neurosurgeons, and radiologic images like head CT are read by radiologists. These services are available 24 hours a day, and head CT is acquired up on presentation of TBI cases. The hospital had an eight-bedded intensive care unit (ICU) for care of critically ill adults and pediatric patients.

### 2.2. Study Design and Period

An observational cross-sectional study was carried out among pediatric TBI cases, aged between 2 months and 14 years and that visited HUCSH from September 2017 to September 2018. Consecutively, admitted TBI cases fulfilling the predefined criteria were included in the study after informed consent was obtained from the family or guardians. Cases were excluded from the study when consent was not secured.

### 2.3. Sample Size

The study was planned for one year with an assumption of getting a minimum sample size of 221 as calculated from a similar study with P, 17.4% [30]. During the one-year study period, 317 TBI cases were observed (95% CI: 314–320) and all were included in the study.

### 2.4. Variables

The dependent variable was a patient's outcome on discharge, i.e., full recovery, neurologic deficit, or death. Poor outcome was defined as death or neurologic disability and was assessed up on hospital discharge of TBI cases. Independent variables included sociodemographic data (age, sex, and place of residence), intent, mechanism and nature of injury, place of occurrence, severity of injury, associated extracranial injury, investigation and treatment type, and duration of hospital stay.

### 2.5. Operational Definitions

Traumatic brain injury (TBI) was defined as a brain injury that occurs following a blow to the head, a fall, a bullet, a high-speed crash, or explosion injuries [1]. TBI severity was graded into mild, moderate, and severe when the GCS was 13–15, 9–12, and ≤8, respectively [6]. Outcome was assessed using the GCS during discharge from the hospital [21, 22]. Hypotension and hypertension were considered when blood pressure for age and sex was below the 5^th^ percentile and ≥95^th^ percentile, respectively [31]. Hypoglycemia and hyperglycemia were considered when the admission random blood sugar was <70 mg/dl and >200 mg/dl, respectively [32, 33].

### 2.6. Data Collection Tool, Procedure, and Data Quality Assurance

Data were collected at the pediatric emergency room, inpatient ward, and intensive care unit by trained Bachelor of Science graduate nurses and intern doctors. Data were collected using a structured questionnaire, and information consisting of sociodemographic characteristics, characteristics of injury (i.e., intent, mechanism, nature, and place of injury), patient's previous medical history, clinical workup and management, duration of admitted stay, and discharge outcome were collected. All consecutive TBI cases seen at the pediatric emergency department of HUCSH were included after consent was obtained from the family or guardian. Daily collected data were checked for completeness by the supervisor and resident doctor, and correction was made on the spot and on a daily basis.

### 2.7. Data Analysis

Data were double-entered into excel spread sheets and analyzed using SPSS version 20 software. Mean and standard deviations were used to present continuous variables. Frequency and percentages were used to present categorical variables. After checking for normality and other parameters, parametric tests were chosen for analysis to assess associations between variables and outcome. Binary logistic regression was used to assess the association between dependent and independent variables. Variables with *P* value < 0.05 on binary logistic regression analysis were taken into multiple logistic regression models for controlling the possible effect of confounders to ascertain associations. Finally, variables which had an independent association with outcome of TBI were identified on the basis of the adjusted odds ratio (aOR), with 95% CI and *P* value < 0.05.

## 3. Results

### 3.1. Sociodemographic Characteristics

There were 4,258 pediatric emergency room (ER) visits, and TBI contributed to 7.4% (317) of ER visits in a one-year study period. A total of 317 study subjects were included. Among 317 study subjects, 218 (68.8%) were males and 99 (31.2%) females with a male to female ratio of 2.2 : 1. The study subjects were aged from 7 months to 14 years with a mean age of 7.66 ± 3.82 years. School age groups, 5–10 years, and adolescents, 10–14 years, contributed to 36.9% (117) and 36.3% (115) of TBI, respectively. One hundred and eighty three (57.7%) of the study subjects came from Southern Nations, Nationalities, and People's Regional State (SNNPR) while the rest 134 (42.3%) came from the neighboring Oromia and Ethiopian Somali regional states (see Table 1). Boys were predominantly affected, and they accounted for 65.8%, 68.3%, and 71.3% of TBI in under fives, school aged, and adolescent age groups, respectively. The majority of our study subjects' parents were farmers or small-scale businesses owners, 313 (98.7%).

### 3.2. Pattern and Mechanism of Traumatic Brain Injury

The main cause of TBI was RTA (road traffic accidents), 144 (45.4%) cases. From RTA, the majority were pedestrians, 120 (83.3%), while 24 (16.7%) were occupants in vehicles. The other TBI causes were falls, 104 (32.8%); fighting/violence, 40 (12.6%); animal bite or kick injury, 28 (8.8%); and one case of assault (child maltreatment). Most preschool falls occurred at home 29 (60.7%), while 94.1% (32) of school aged and 97.6% (41) of adolescent falls happened outdoor. Eighty three (79.8%) of falls and 84 (58.3%) of road traffic accidents occurred in boys.

Concerning the timing of presentation, most of the study subjects presented within 24 hours of injury, 258 (81.4%). Thirty one (9.8%) and 28 (8.8%) of TBI cases presented between 24 hours and 72 hours and after 72 hours of injury (see Table 2).

The majority of TBI followed unintentional injuries, 87.4% (277). Almost all of the intentional injuries, 38 (97.5%), were due to fighting with only one case of child maltreatment. Eighty nine (28%) of TBI cases had lost consciousness at the time of presentation. Among the study subjects, 26 (8.2%) exhibited seizure, 40 (12.6%) had abnormal pupillary signs, and 60 (18.9%) showed signs of increased ICP. Hypertension and depressed mentation were the most common manifestations of increased ICP, each accounting for 22 (6.9%) raised ICP cases. Vomiting 16 (5%) was the other commonly raised ICP feature (see Table 2).

Majority of our study subjects were conscious at the time of presentation, and 195 (61.5%) had a GCS of 15/15. The least GCS was 3 in 4 cases, and mean GCS at admission was 13.4 ± 2.7. Concerning TBI severity, mild, moderate, and severe form accounted for 231 (72.9%), 61 (19.2%), and 25 (7.9%) of study subjects, respectively. Mild TBI was caused by all mechanisms of injury, while moderate and severe TBI were mainly caused by RTA. On presentation, hypotension and hyperglycemia were documented in 9 (2.8%) and 20 (6.3%) of TBI cases (see Table 2).

Skull X-ray was done in 177 (55.8%) of study subjects, and skull fractures were reported in 100 (31.5%) TBI cases: linear skull fractures, 27 (8.5%); depressed skull fractures, 72 (22.7%); and basal skull fracture, 1 (0.3%). Head CT scan was done in 70.7% (224) of the study subjects, and intracranial hematomas were documented in 4.4% (14) of the study subjects: 3 epidural, 6 subdural, 4 intracerebral, and 1 subarachnoid hemorrhage. Additional head CT findings included cerebral contusion and diffuse axonal injury, simple skull fractures without intracranial bleeding, and depressed skull fractures with contusion or intracranial bleeding in 30 (9.4%), 94 (29.7%), and 36 (11.4%) of the study subjects, respectively. Normal head CT scan was documented in 50 cases (15.8%) of TBI. Extensive intracerebral hemorrhages, 4 (40%), and diffuse axonal injury (DAI), 4 (40%), were the head CT findings in fatal cases. Head CT was not done in the other two fatal cases (see Table 2).

Associated extracranial injuries were reported in 256 (80.7%) cases of TBI. From these, soft tissue injury was the most common form, 179 (56.5%), followed by extremity bone fracture, 70 (22.1%); chest or abdominal injury, 6 (1.9%); and one case of vertebral bone injury (see Table 3).

### 3.3. Management and Outcome

One hundred twenty nine (72.2%) TBI cases were managed conservatively, while 88 (27.8%) underwent various surgical interventions within the first (01) week of presentation. Most of the operated cases were aged 5 to 10 years, 48/88 (55%). No death was documented among the operated patients. The common surgical indications were the evacuation of epidural and subdural hematoma, 4 (4.5%); wound debridement for compound skull fracture, 19 (21.3%); and depressed skull fracture elevation, 66 (74.2%) (see Table 3). Elevation of depressed skull fracture was done for 66 cases with mild (53), moderate (12), and severe (1) TBI categories. Burr hole and evacuation was done for four mild TBI cases. Irrigation and debridement were done for mild (16) and moderate TBI (1) cases.

Concerning the hospital stay, majority of the TBI cases stayed from 4 to 7 days, 34% (108), while 78 (24.6%) were discharged within 24 hours of arrival. Prolonged hospitalization (≥1 month) was documented in 2 patients with severe TBI and extremity fracture with extensive soft tissue injury. The average length of hospitalization days among those who died was 4.5 days (median 2 days), and 5/10 (50%) of deaths occurred within the first 3 days of admission (see Table 3).

Concerning the outcome at discharge, 303 (95.6%) study subjects recovered from the injury. Good recovery without neurologic deficit, focal neurologic deficit, and depressed mentation were documented in 267 (84.2%), 30 (9.5%), and 10 (3.2%) of study subjects, respectively. Ten (3.2%) deaths were documented, and 2 cases were referred to another hospital and 2 left against medical advice (see Table 3).

### 3.4. Factors Affecting Outcome

On bivariate analysis, factors significantly associated with a poor outcome of pediatric TBI with 95% CI and *P* value < 0.05 were comorbid illness, loss of consciousness and convulsion at presentation, increased ICP sign, severity of head injury, presence of hypotension, hyperglycemia on presentation, and head CT scan findings.

On multivariable logistic regression, presence of increased ICP at admission was associated with 1.4 times the odds of death or neurologic disability [AOR: 1.415 (95% CI: 1.458–9.557)]. Severe TBI was associated with doubled odds of death or disability compared with moderate and mild TBI [AOR: 2.553 (95% CI: 1.965–4.524)]. Presence of hyperglycemia [AOR: 2.318 (95% CI: 1.873–7.874)] and contusion, DAI, or intracranial bleeding on head CT [AOR: 2.45 (95% CI: 1.811–7.952)] were also found to be significantly associated with death or neurologic disability among pediatric TBI (see Table 4).

## 4. Discussion

In our study, boys and children above 5 years of age were predominantly affected by TBI. This finding is in agreement with Nigerian, South African, and Tunisian studies. This could be related to boy's risk-prone behavior resulting in high energy transfer and their outdoor engagement [14, 30, 34–37]. Concerning mechanisms of injury, unintentional pedestrian RTA and falls were the most common causes followed by intentional fights/violence injuries. This is in line with most of the studies done in developing countries [2, 8, 18, 22, 30, 34, 37–39].

Most of our study subjects presented within 24 hours of injury and had a mild form of TBI. This is in harmony with reports from Nigeria and Tunisia [30, 34, 38, 40]. However, severe TBI was the most common form in other Nigerian and Tunisian studies. The possible reason for the noted difference could be the exclusion of mild TBI cases not meeting the admission criteria in the Nigerian study as well as all studied subjects in the Tunisian study were ICU (intensive care unit) admitted patients with severe TBI or severity feature [30, 39]. Our study subjects had hypotension, lost consciousness, convulsion, and increased intracranial pressure sign at presentation. Comparable reports were documented in other studies [30, 37].

In this study, subjects were evaluated with random blood sugar, skull X-ray, and head CT scan at presentation. Most of the subjects had normal glucose levels with hyperglycemia documented in 6.3% of TBI cases. Head CT scan was ordered in 70.7% of study subjects and showed various types of skull bone fractures, brain contusion, intracranial bleeding, and diffuse axonal injury. These findings are in agreement with studies done in India, Tunisia, and Nigeria, and head CT scan requests conform to the recommended neurosurgical practice [22, 25, 30, 37, 39].

In our study, associated extracranial injuries were documented in 80% of the study subjects with soft tissue injury being the most common followed by extremity bone fracture. Reports from Nigeria and Nepal documented similar findings [18, 40]. Most of the subjects in our study were managed conservatively (72.2%), while neurosurgical interventions like depressed skull elevation, irrigation and debridement, and hematoma evacuation were common procedures. Studies from India, South Africa, Nigeria, and Tunisia had documented similar findings [37–39, 41]. Concerning the length of hospital stay, majority of our study subjects stayed from 4 to 7 days while nearly a quarter (24.6%) were discharged in the first 24 hours of admission. Shorter hospital stays could be due to the higher percentage of milder forms of TBI in our study. These findings are comparable to similar studies done in Africa and China [22, 29, 41, 42]. Moreover, a detailed review and discussion on the challenges in the surgical and medical management of severe and penetrating TBI suggested a more conservative approach envisioning for better options and outcomes in the future [43].

In this study, good functional outcome (recovery without any neurologic deficit) was documented in 84.2% (95% CI, 80.2–88.1) of study subjects. This is higher than the Indian and Chinese studies [37, 42]. It is lower than the Nigerian and South African studies [34, 39, 41]. Ten (3.2%, 95% CI: 1.5–5.7%) of our study subjects died. This is comparable with the South African report [41], but it is lower than the Chinese and Nigerian studies [34, 37, 38, 40, 42]. Differences in outcome could be explained by the health facilities capacities, severity and mode of head injury, and age of inclusion as the above Nigerian study included children up to 18 years of age. Additionally, the majority of our study subjects had a milder form of TBI.

In the current study, severe TBI was associated with doubled odds of death/neurologic disability, when compared with mild and moderate head injuries. This finding is in harmony with the Nigerian and Indian studies [39, 40, 44]. Severe TBI is associated with primary is are flexia and secondary brain edema, which predict the outcome of the patient [45]. Head CT scan findings of contusion, diffuse axonal injury, and intracranial bleedings were associated with 2.45 times higher chances of death or neurologic disability when compared with normal head CT cases. This is in agreement with the Tunisian and Nigerian studies [22, 30, 39]. Diffuse axonal injury that occurs in TBI is reported to be secondary to axonal swelling, calcium-mediated irreversible blockade of axonal transport, swollen endoplasmic reticulum, etc. Intracerebral, brainstem, intraventricular and corpus callosum hemorrhages, and bleeding near the third ventricle on head CT are reported evidences for DAI. It is reported that these findings predict poor outcomes among TBI cases with the highest accuracy [46–48].

Our study documented 1.42 times higher chances of death and neurologic disability among TBI cases who presented with ICP signs when compared with those without ICP signs. This is in line with the Tunisian and Argentinian studies [22, 30, 49]. Glial swelling with narrowed lumina of the microvasculature due to podocytic process swelling results in increased ICP. Diffuse cerebral ischemia also results in calcium hemostatic imbalance and activation of anaerobic metabolism. Increased ICP clinically presents as hypoxemia, seizure, mental level deterioration, and neurologic deficits [50, 51].

In this study, admission hyperglycemia was associated with 2.32 times higher chances of death and neurologic disability. Similar findings were reported in studies conducted in Turkey and Singapore [32, 52, 53]. Impaired cerebral mitochondrial dysfunction following TBI is thought to result in hyperglycemia [54, 55], though detailed mechanisms are yet to be studied [56]. We did not measure increased intracranial pressure by invasive methods as devices and facilities were not readily available, and this is the major limitation of our study, as the practice of these and related neuromonitoring methods for TBI are currently recommended [57, 58]. Secondly, long-term follow-up on neurological outcomes was not determined as the outcome was assessed on hospital discharge.

## 5. Conclusion

In our study, boys and children above 5 years of age were highly affected by TBI. Pedestrian RTA, falls, fights, and animal-related injuries with milder form and early presentation were the most common mechanisms of injuries. Various forms of skull vault fractures, hemorrhage, contusion, and axonal injuries were documented on the head CT scan. The majority of our study subjects were managed conservatively and recovered without neurologic deficits. Death was documented in 10 (3.2%) of the study subjects. Increased ICP, hyperglycemia, severe TBI, and contusion, diffuse axonal injury, or intracranial bleeding on head CT were predictors for death or neurological disability among pediatric cases of TBI. Strategies to ensure road safety and to prevent falls and animal-related injuries, closer follow-up of TBI cases for ICP, and proper glycemic controls are recommended.

## Figures and Tables

**Table 1:** Sociodemographic characteristics of pediatric traumatic brain injury at HUCSH from September 2017 to September 2018.

Variable	Category	Number (*n*)	Percentage (%)
Age (years)	<5	85	26.8
	5–10	117	36.9
	10–14	115	36.3

Gender	Male	218	68.8
	Female	99	31.2

Parental occupation	Employee	75	23.7
	Merchant	65	20.5
	Farmer	173	54.6
	Driver	2	0.6
	Other	2	0.6

Place of residence	Hawassa city	112	35.3
	Oromia	133	42
	Other SNNPR	71	22.4
	Somali	1	0.3

Chronic illness	No	313	98.7
	Severe acute malnutrition	2	0.6
	Epilepsy	1	0.3
	Other	1	0.3

**Table 2:** Pattern and mechanism of pediatric traumatic brain injury at HUCSH from September 2017 to September 2018.

Characteristics of traumatic brain injury	Subclassification	*N* (%)
Mechanism of injury	Road traffic accident	144 (45.4)
	Falls	104 (32.8)
	Fighting	40 (12.6)
	Animal kick or bite	28 (8.8)
	Assault/child abuse	1 (0.3)

Time of arrival after injury	<24 hours	258 (81.4)
	1–3 day	31 (9.8)
	>3 day	28 (8.8)

Place of occurrence	Home	29 (9.1)
	Outdoor	144 (45.4)
	Occupant in vehicle	24 (7.6)
	Pedestrian	120 (37.9)

Loss of consciousness at presentation	No	228 (71.9)
	Yes	89 (28.1)

Convulsion at presentation	No	291 (91.8)
	Yes	26 (8.2)

Signs of increased ICP^*∗*^	No	257 (81.1)
	Yes	60 (18.9)

Sign and symptom of increased ICP	Vomiting	16 (5.1)
	Hypertension	22 (6.9)
	Decreased mentation	22 (6.9)

Pupillary sign	Unilaterally fixed	22 (6.9)
	Symmetrically fixed	3 (0.9)
	Midsized and reactive	277 (87.4)
	Bilaterally dilated	15 (4.8)

Severity of TBI^*∗∗*^	Mild TBI	231 (72.9)
	Moderate TBI	61 (19.2)
	Severe TBI	25 (7.9)

Head CT and scan finding	Normal	50 (15.8)
	Skull fracture	94 (29.7)
	DSF^*∗∗∗*^ with contusion, DAI+, intracranial bleeding	36 (11.4)
	Contusion/DAI	30 (9.5)
	Intracranial bleeding	14 (4.4)
	Not done	93 (29.3)

Skull X-ray finding	Normal	77 (24.3)
	Linear skull fracture	27 (8.5)
	Depressed skull fracture	72 (22.7)
	Not done	141 (44.5)

Hypotension on admission	No	308 (97.2)
	Yes	9 (2.8)

Hyperglycemia on admission	No	297 (93.7)
	Yes	20 (6.3)

^*∗*^Intracranial pressure. ^*∗∗*^Traumatic brain injury. ^*∗∗∗*^Depressed skull fracture, +diffuse axonal injury. CT: computed tomography

**Table 3:** Management and outcome of pediatric traumatic brain injury at HUCSH from September 2017 to September 2018.

Head injury characteristics	Subclassification	*N* (%)
Associated extracranial injury	No injury	61 (19.2)
	Soft tissue injury	179 (56.5)
	Extremity bone fracture	70 (22.1)
	Chest/abdominal injury	6 (1.9)
	Vertebral bone fracture	1 (0.3)

Management type	Surgical	88 (27.8)
	Conservative	229 (72.2)

Type of surgical intervention	Burr hole	2 (0.6)
	Elevation for depressed skull fracture	66 (20.8)
	Craniotomy and evacuation	2 (0.6)
	Irrigation and debridement	19 (6.0)

Condition on discharge	Survived/recovered	303 (95.6)
	Died	10 (3.2)
	Referred	2 (0.6)
	Left against medical advice	2 (0.6)

Neurologic outcome at discharge	No deficit	267 (84.2)
	Focal deficit	30 (9.5)
	Depressed mentation	10 (3.2)

Duration of hospital stay	≤24 hours	78 (24.6)
	1–3 days	64 (20.2)
	4–7 days	108 (34.1)
	8 days–01 month	65 (20.5)
	≥1 month	2 (0.6)

**Table 4:** Multivariable logistic regression analysis of factors associated with pediatric traumatic brain injury outcome at HUCSH (*n* = 317).

Variables	Categories	No. (%)	COR^*∗*^ (95% CI)	AOR^*∗∗*^ (95% CI)	*P* value
Hyperglycemia at presentation	No	297 (93.7)	1	1	*p*=0.003
	Yes	20 (6.3)	4.461 (1.855–10.728)	2.318 (1.873–7.874)	

Increased ICP^+^ sign	No	257 (81.1)	1	1	*P*=0.002
	Yes	60 (18.9)	2.757 (1.951–3.895)	1.415 (1.4058–9.557)	

Severity of TBI^#^ at presentation	Mild TBI	231 (72.9)	1	1	*P*=0.029
	Moderate	61 (19.2)	1.904 (1.547–2.343)	2.553 (1.965–4.524)	
	Severe	25 (7.9)			

CT% scan finding	Normal or skull fracture	134 (45.5)	1	1	*P*=0.005
	Contusion, DAI^^^, or ICH^$^	80 (25.3)	2.061 (1.635–2.599)	2.45 (1.811–7.952)	

Loss of consciousness at presentation	No	228 (71.9)	1	1	*P*=0.168
	Yes	89 (28.1)	1.844 (1.523–2.246)	1.271 (0.874–2.116)	

Hypotension at presentation	No	308 (97.2)	1	1	*P*=0.259
	Yes	9 (2.8)	2.591 (1.027–6.534)	1.629 (0.301–5.101)	

Convulsion at presentation	No	291 (91.8)	1	1	*P*=0.816
	Yes	26 (8.2)	3.344 (1.773–6.307)	1.244 (0.296–0.902)	

Comorbid illness	No	313 (98.7)	1	1	*P*=0.873
	Yes	4 (1.3)	1.706 (1.64–4.551)	1.325 (0.157–2.124)	

COR^*∗*^, crude odd ratio; AOR^*∗∗*^, adjusted odds ratio; ICP^+^, intracranial pressure; TBI^#^, traumatic brain injury; CT%, computed tomography; DAI^^^, diffuse axonal injury; ICH^$^, intracranial hemorrhage.

## Data Availability

The datasets analyzed during this study are available from the corresponding author on reasonable request.
